# Diet quality in older age: the influence of childhood and adult socio-economic circumstances

**DOI:** 10.1017/S0007114515000604

**Published:** 2015-04-01

**Authors:** Janice L. Atkins, Sheena E. Ramsay, Peter H. Whincup, Richard W. Morris, Lucy T. Lennon, S. Goya Wannamethee

**Affiliations:** 1 Department of Primary Care and Population Health, University College London Medical School, Royal Free Campus, LondonNW3 2PF, UK; 2 Division of Population Health Sciences and Education, Population Health Research Centre, St George's University of London, LondonSW17 0RE, UK

**Keywords:** Diet quality, Diet score, Older adults, Social relationships, Socio-economic factors

## Abstract

Socio-economic gradients in diet quality are well established. However, the influence of material socio-economic conditions particularly in childhood, and the use of multiple disaggregated socio-economic measures on diet quality have been little studied in the elderly. In the present study, we examined childhood and adult socio-economic measures, and social relationships, as determinants of diet quality cross-sectionally in 4252 older British men (aged 60–79 years). A FFQ provided data on daily fruit and vegetable consumption and the Elderly Dietary Index (EDI), with higher scores indicating better diet quality. Adult and childhood socio-economic measures included occupation/father's occupation, education and household amenities, which combined to create composite scores. Social relationships included social contact, living arrangements and marital status. Both childhood and adult socio-economic factors were independently associated with diet quality. Compared with non-manual social class, men of childhood manual social class were less likely to consume fruit and vegetables daily (OR 0·80, 95 % CI 0·66, 0·97), as were men of adult manual social class (OR 0·65, 95 % CI 0·54, 0·79), and less likely to be in the top EDI quartile (OR 0·73, 95 % CI 0·61, 0·88), similar to men of adult manual social class (OR 0·66, 95 % CI 0·55, 0·79). Diet quality decreased with increasing adverse adult socio-economic scores; however, the association with adverse childhood socio-economic scores diminished with adult social class adjustment. A combined adverse childhood and adulthood socio-economic score was associated with poor diet quality. Diet quality was most favourable in married men and those not living alone, but was not associated with social contact. Diet quality in older men is influenced by childhood and adulthood socio-economic factors, marital status and living arrangements.

Diet is a crucial modifiable risk factor for morbidity and mortality^(^ ^1^ ^–^ ^3^ ^)^. A healthy diet is especially important in an elderly population who are at an increased risk of chronic disease, especially CVD, and it is therefore important to understand the determinants of dietary intake in the elderly^(^ ^1^ ^,^ ^4^ ^)^. Historically, dietary research has focused on single food items and nutrients; however, in recent times, this focus has shifted to total diet quality and dietary patterns, to reflect the fact that foods are not eaten in isolation and there may be interactions between foods or nutrients consumed^(^ ^5^ ^,^ ^6^ ^)^. Many predefined dietary scores have been developed based on adherence to dietary recommendations or specific dietary patterns^(^ ^7^ ^,^ ^8^ ^)^, with the Mediterranean Diet Score being one of the most commonly used markers of overall diet quality^(^ ^9^ ^,^ ^10^ ^)^.

Strong socio-economic gradients in diet quality are well established, and studies have shown consistently that higher socio-economic groups are more likely to consume a Mediterranean-style diet, characterised by a high consumption of fresh fruit and vegetables, whole grains, lean meats, fish and low-fat dairy products^(^ ^11^ ^–^ ^14^ ^)^. A meta-analysis of 18- to 85-year-olds from seven European countries has also shown strong associations between higher socio-economic position (SEP), defined using occupation or education, and a greater daily fruit and vegetable consumption^(^ ^15^ ^)^. It has been suggested that not only adult socio-economic factors, but also childhood socio-economic factors, may have an impact on adult dietary patterns^(^ ^16^ ^,^ ^17^ ^)^. Apart from socio-economic factors, social relationships such as frequency of contact, living arrangements and marital status, which are known to be associated with increased mortality and CVD risk^(^ ^18^ ^)^, are also increasingly being recognised as important dietary determinants particularly in the older population^(^ ^19^ ^,^ ^20^ ^)^. Although socio-economic gradients in diet quality have been shown to persist in the older population^(^ ^21^ ^–^ ^25^ ^)^, the influence of different types of material socio-economic conditions particularly in childhood, and the use of multiple disaggregated socio-economic measures on diet quality have been little studied in the elderly.

The present study aimed to examine the associations between a range of childhood and adult socio-economic measures (including occupation/father's occupation, education and household amenities) and social relationships (social contact, living arrangements and marital status) with diet quality in older British men. Daily fruit and vegetable consumption and the Elderly Dietary Index (EDI) were used as markers of a Mediterranean-style diet and hence of overall diet quality^(^ ^26^ ^)^.

## Methods

### Study population

The British Regional Heart Study (BRHS) is a prospective study of CVD, in a socio-economically and geographically representative sample of 7735 men, drawn from general practices in twenty-four towns across Great Britain^(^ ^27^ ^)^. The cohort was initially examined in 1978–80 and is predominantly of white European ethnic origin (>99 %). In 1998–2000, 4252 men aged 60–79 years (77 % of survivors) attended a 20th-year re-examination. All men attended a physical examination, provided a fasting blood sample, and completed a lifestyle questionnaire and a FFQ^(^ ^28^ ^)^. The present study is a cross-sectional analysis using data from the 20th-year re-examination and additional data on childhood and adult social circumstances from questionnaires. All participants provided written informed consent, in accordance with the Declaration of Helsinki. Ethical approval was obtained from relevant local research ethics committees.

### Dietary assessment

In 1998–2000, participants completed a self-administered postal FFQ that was used to assess usual weekly consumption of eighty-six food and drink items. The FFQ was developed for use in the WHO's Monitoring Trends and Determinants in Cardiovascular Disease Survey^(^ ^29^ ^)^, and has been validated in the British population against weighed food intake^(^ ^30^ ^,^ ^31^ ^)^. Participant's total energy intakes were all within a range compatible with a normal lifestyle (2092–33 472 kJ/d (500–8000 kcal/d) in men^(^ ^32^ ^)^), so no exclusions were made on this basis.

We examined diet quality using a predefined dietary score, the EDI, as a marker of a Mediterranean-style diet. The EDI score was used instead of the Mediterranean Diet Score as it was developed specifically to address adherence to nutritional recommendations for older adults^(^ ^26^ ^,^ ^33^ ^)^, and it has also previously shown the strongest association with CVD mortality and all-cause mortality, compared with other dietary scores, in this population^(^ ^34^ ^)^. The EDI differs from the Mediterranean Diet Score as it uses a four-point scoring system for each food component as opposed to a dichotomous scoring system, and the EDI score used here does not include alcohol intake. The EDI consisted of nine food components (meat, fish and seafood, vegetables, cereals, fruit, legumes, olive oil, dairy products, and bread), each scoring 1–4 based on the frequency of consumption, with a total score ranging from 9 to 36. Additional details pertaining to the scoring of the EDI are included in online supplementary Table S1. Higher scores of the EDI indicated greater adherence to dietary recommendations and a healthier diet quality. Participants were categorised into four ordered groups of the EDI score, which were as close to quartiles as possible.

In addition to the EDI, daily fruit and vegetable consumption was used as an additional marker of diet quality. Fruit and vegetable consumption, which has consistently been shown to be strongly associated with reduced CVD mortality^(^ ^35^ ^)^, is simpler to measure than the EDI and hence less prone to measurement error. Participants were asked how frequently they consumed fresh fruit and vegetables (1, 2, 3, 4, 5, 6 or 7 d/week, monthly, or rarely/never). Daily consumption was classified as both fruit and vegetable consumption on 7 d/week.

### Measures of socio-economic position

Adult occupational social class was based on the longest-held occupation recorded at study entry (aged 40–59 years) using the Registrar General's occupational classification: I (professionals), II (managerial), III non-manual (semi-skilled non-manual), III manual (semi-skilled manual), IV (partly skilled) and V (unskilled). Participants were classified as manual or non-manual, excluding men who were in the Armed Forces (*n* 112). Additional socio-economic measures available from questionnaires were education (age at leaving full-time education), pension (state only or state plus private pension), car and house ownership, and whether participants had central heating at home. A composite score combining adverse socio-economic measures was created to investigate the cumulative impact of low SEP, and to take into account a range of socio-economic measures that may have a greater impact than occupation alone. One point was assigned for adult manual social class, education ≤ 14 years, no car, not a house owner, state pension only and no central heating, to generate a total score ranging from 0 to 6^(^ ^36^ ^)^.

Childhood socio-economic measures were collected through a postal questionnaire in 1992. Childhood occupational social class was based on father's longest-held occupation. Participants were classified as manual or non-manual using the Office of Population Censuses and Surveys Classification of Occupations (1980) social class coding manual^(^ ^37^ ^)^. Men whose father's longest-held occupation was the Armed Forces were excluded (*n* 81). Information was also collected on childhood household amenities. Participants were asked whether, up to 10 years old, their home had a bathroom, hot water supply or family car ownership. An adverse childhood socio-economic score was created, as a marker of overall early-life SEP, including childhood manual social class, no bathroom, no hot water supply and no family car ownership, to generate a total score ranging from 0 to 4^(^ ^36^ ^)^.

To assess the combined effect of childhood and adult social class on diet quality, participants were categorised into four combined childhood and adult social class groups: childhood and adult non-manual social class; childhood non-manual and adult manual social class; childhood manual and adult non-manual social class; childhood and adult manual social class. In addition, a combined adverse childhood and adulthood socio-economic measure score was created by summing the adverse adult and childhood scores, to generate a total score ranging from 0 to 10.

### Measures of social relationships

In the 1998–2000 questionnaire, men were asked how often they saw or spoke to their children, siblings, friends and neighbours (every week, every month, every few months, every year, rarely/never and does not apply), whether they were living alone (living alone, living with a partner/spouse, living with other family members and living with other people), and about their marital status (single, married, widowed, divorced/separated and other). Participants whose marital status was classified as ‘other’ were excluded from the analysis (*n* 8).

### Covariates

Information on cigarette smoking, alcohol intake and physical activity were collected via a questionnaire in 1998–2000. Men were classified into four smoking groups (never smoked, long-term ex-smokers (>15 years), recent ex-smokers ( ≤ 15 years) and current smokers)^(^ ^38^ ^)^. Alcohol intake was classified into five groups based on the number and frequency of alcoholic beverages consumed (none, occasional, light, moderate and heavy)^(^ ^39^ ^)^. Current physical activity was classified into six groups based on exercise frequency and intensity (inactive, occasional, light, moderate, moderately vigorous and vigorous)^(^ ^40^ ^)^. Height and weight were measured at the 20th-year re-examination. BMI was calculated, and participants were classified into four WHO-defined groups (underweight < 18·5 kg/m^2^, normal weight 18·5–24·99 kg/m^2^, overweight 25–29·99 kg/m^2^ and obese ≥ 30 kg/m^2^)^(41)^.

### Statistical analysis

Of the 4252 men attending the 20th-year re-examination, data on fruit and vegetable intake were available for 4067 participants; of these, 3924 men had adequate data to generate the EDI score. Descriptive characteristics of the participants were presented by EDI quartiles and by daily fruit and vegetable intake. *P* values for trend across EDI quartiles were obtained using regression analyses, and *P* values for difference between groups for daily fruit and vegetable intake were obtained using χ^2^ tests. The relationship between childhood and adult socio-economic measures was assessed using correlation coefficients. Multivariable logistic regression assessed the associations between childhood and adult socio-economic measures, and social relationships, with daily fruit and vegetable intake and being in the top quartile of the EDI. OR for the EDI were presented for being in the highest quartile compared with the lower three quartiles. Logistic regression models were adjusted for age, energy intake (kJ/d), smoking status, alcohol intake, physical activity and BMI. In addition, childhood socio-economic measures and social relationships were adjusted for adult occupational social class, and adult socio-economic measures were adjusted for childhood occupational social class. For the adjustments, age and energy intake were fitted as continuous variables, and smoking status, alcohol intake, physical activity, BMI, adult and childhood social class were fitted as categorical variables. A test for interaction between childhood and adult social class was also carried out. All analyses were performed in Stata 12.1 (Stata Corporation).

## Results

Only 17·9 % of participants consumed fresh fruit and vegetables daily. The EDI score was normally distributed with a mean of 24·2, an sd of 3·3, ranging from 12 to 35. Table 1 presents the cohort characteristics by EDI quartiles and by daily fruit and vegetable intake. In the highest EDI quartile, there was a significantly lower proportion of men who were current smokers, heavy drinkers, physically inactive, of manual adult social class and of manual childhood social class, and had a slightly lower mean age and total energy intake. In those who consumed fruit and vegetables daily, there was a significantly lower energy intake and a significantly lower proportion of current smokers, physically inactive, manual adult social class and manual childhood social class. The correlations between socio-economic measures in childhood and adulthood are summarised in Table 2. Overall, correlations were modest but significant, with the strongest correlations found between childhood measures and adult occupational social class and education.

**Table 1:** Characteristics of the British Regional Heart Study participants aged 60–79 years by diet quality (Elderly Dietary Index (EDI) quartiles and daily fruit and vegetable intake) (Mean values and standard deviations; number of participants and percentages)

	EDI quartiles*		Daily fruit and vegetable intake†	
1st (12–22 points)	2nd (23–24 points)	3rd (25–26 points)	4th (27–35 points)		No	Yes	
	Mean	sd	Mean	sd	Mean	sd	Mean	sd	*P*	Mean	sd	Mean	sd	*P*
*n*	1074	982	901	967		3338	729	
Age (years)	68·8	5·5	68·6	5·5	68·6	5·6	68·1	5·3	0·007	68·6	5·5	68·8	5·4	0·54
Energy intake									< 0·001					0·001
kJ/d	8964·2	2404·5	9127·8	2239·7	8950·0	2218·4	8501·9	1854·3		8871·8	2248·1	8789·0	2033·8	
kcal/d	2142·5	574·7	2181·6	535·3	2139·1	530·2	2032	443·2		2120·4	537·3	2100·6	486·1	
Current smokers									< 0·001					< 0·001
*n*	265	114	67	45		473	36	
%	24·7	11·7	7·4	4·7		14·2	5·0	
Heavy drinkers									< 0·001					0·26
*n*	43	30	22	12		97	16	
%	4·2	3·1	2·5	1·3		3·0	2·2	
Physically inactive									< 0·001					0·04
*n*	158	99	95	80		385	66	
%	15·4	10·4	10·8	8·6		12·0	9·3	
Obese (BMI >30 kg/m^2^)									0·110					0·95
*n*	190	180	162	145		575	125	
%	17·8	18·4	18·1	15·1		17·3	17·2	
Adult manual social class									< 0·001					< 0·001
*n*	687	518	374	357		1757	279	
%	66·0	54·1	42·6	38·2		54·1	39·7	
Childhood manual social class									< 0·001					< 0·001
*n*	745	637	565	545		2171	422	
%	78·2	72·7	68·1	60·9		72·2	63·3	

* Data for the EDI available for 3924 participants. *P* for trend across the EDI quartiles.

† Data for fruit and vegetable intake available for 4067 participants. *P* for difference between groups.

**Table 2:** Correlations between childhood and adult socio-economic measures

	Childhood socio-economic measures
Adult socio-economic measures	Childhood social class	Bathroom	Hot water supply	Family car ownership
Adult social class	0·25**	0·18**	0·20**	0·17**
Education	0·22**	0·21**	0·23**	0·19**
Car ownership	0·09**	0·10**	0·10**	0·10**
House ownership	0·09**	0·07**	0·08**	0·06**
Pension	0·12**	0·09**	0·09**	0·05*
Central heating	0·05*	0·04*	0·05*	0·05*

* *P*< 0·05.

** *P*< 0·001.

Table 3 presents the OR of being in the top quartile of the EDI and of consuming fruit and vegetables daily according to childhood socio-economic measures. Childhood social class was the strongest childhood socio-economic measure associated with diet quality, with men of manual childhood social class being significantly less likely to be in the top EDI quartile (OR 0·73, 95 % CI 0·61, 0·88) and to consume fresh fruit and vegetables daily (OR 0·80, 95 % CI 0·66, 0·97), independent of behavioural factors and adult social class. In sensitivity analysis, further adjusting for all the other adult socio-economic measures, the associations were attenuated slightly between childhood social class and the EDI (OR 0·81, 95 % CI 0·67, 0·98) and daily fruit and vegetable consumption (OR 0·82, 95 % CI 0·66, 1·02). Men with no family car ownership in childhood were less likely to be in the highest EDI quartile, with borderline significance after adjustment for behavioural factors and adult social class, but family car ownership was not significantly associated with fruit and vegetable intake. Whether the childhood home had a bathroom or a hot water supply showed no significant associations with either the EDI or daily fruit and vegetable consumption. There was a significant trend between the adverse childhood socio-economic score and the EDI, but no significant trend with daily consumption of fruit and vegetables after adjustment for behavioural factors and adult social class.

**Table 3:** Top quartile of the Elderly Dietary Index (EDI) and daily fruit and vegetable intake according to childhood socio-economic (SE) measures (Odds ratios and 95 % confidence intervals)

	EDI quartiles*	Daily fruit and vegetable intake†
			Unadjusted	Adjusted‡			Unadjusted	Adjusted‡
	*n*	% Q4	OR	95 % CI	*P*	OR	95 % CI	*P*	*n*	%	OR	95 % CI	*P*	OR	95 % CI	*P*
Childhood social class																
Non-manual	1062	33·0	1			1			1081	22·7	1			1		
Manual	2492	21·9	0·57	0·49, 0·67	< 0·001	0·73	0·61, 0·88	0·001	2593	16·3	0·66	0·56, 0·79	< 0·001	0·80	0·66, 0·97	0·03
Childhood household amenities																
Bathroom																
Yes	1906	27·2	1			1			1960	19·1	1			1		
No	1820	22·9	0·79	0·68, 0·92	0·002	0·95	0·81, 1·12	0·57	1895	17·0	0·87	0·74, 1·02	0·09	0·95	0·80, 1·14	0·61
Hot water supply																
Yes	1955	27·7	1			1			2013	20·0	1		0·001	1		
No	1768	22·3	0·75	0·64, 0·87	< 0·001	0·94	0·80, 1·12	0·51	1838	16·0	0·76	0·64, 0·90	0·001	0·85	0·71, 1·02	0·08
Family car ownership																
Yes	617	31·9	1			1			630	19·8	1			1		
No	3110	23·8	0·66	0·55, 0·80	< 0·001	0·81	0·66, 1·00	0·05	3227	17·7	0·87	0·70, 1·08	0·20	1·01	0·80, 1·27	0·95
Adverse childhood SE score§																
0	356	33·7	1			1			361	19·1	1			1		
1	570	32·8	0·96	0·73, 1·27	< 0·001	1·11	0·82, 1·51	0·03	578	24·7	1·39	1·01, 1·92	< 0·001	1·42	1·01, 1·99	0·12
2	845	22·6	0·57	0·44, 0·75		0·78	0·57, 1·05		885	16·4	0·83	0·60, 1·14		1·01	0·72, 1·42	
3	479	25·7	0·68	0·50, 0·92		0·93	0·67, 1·29		491	21·4	1·15	0·82, 1·62		1·33	0·92, 1·91	
4	1281	21·1	0·53	0·41, 0·68		0·78	0·58, 1·05		1333	15·0	0·75	0·55, 1·01		0·93	0·66, 1·29	

* Data for the EDI available for 3924 participants. OR for quartile 4 *v.* quartiles 1–3.

† Data for fruit and vegetable intake available for 4067 participants.

‡ Adjusted for age, energy intake, smoking status, physical activity, alcohol intake, BMI and adult social class.

§ Score includes childhood manual social class, no bathroom, no hot water supply and no family car ownership.

The OR of being in the top quartile of the EDI and consuming fruit and vegetable daily according to adult socio-economic measures are given in Table 4. Adult social class was strongly associated with diet quality, with men of manual social class being significantly less likely to be in the top EDI quartile (OR 0·66, 95 % CI 0·55, 0·79) and to consume fresh fruit and vegetables daily (OR 0·65, 95 % CI 0·54, 0·79), independent of behavioural factors and childhood social class. Additional sensitivity analysis, further adjusting for all the other adult socio-economic measures, showed that the associations were attenuated between adult social class and the EDI (OR 0·86, 95 % CI 0·70, 1·06) and daily fruit and vegetable consumption (OR 0·74, 95 % CI 0·59, 0·93). Examining occupational social class as a continuous variable showed that for every unit decrease in social class, the odds of being in the top quartile of the EDI (OR 0·82, 95 % CI 0·77, 0·88) and of consuming fruit and vegetables daily (OR 0·81, 95 % CI 0·75, 0·88) decreased. Men with a state pension only or ≤ 14 years of education were significantly less likely to be in the highest EDI quartile and to consume fruit and vegetables daily. Examining education as a continuous variable showed that for every additional year of education, the odds of being in the top quartile of the EDI (OR 1·03, 95 % CI 1·01, 1·05) and of consuming fruit and vegetables daily (OR 1·02, 95 % CI 1·01, 1·04) increased. In addition, men who were not car owners, not house owners or did not have central heating were significantly less likely to be in the highest EDI quartile, but these variables were not associated with daily fruit and vegetable consumption. There was a significant inverse trend between the adverse socio-economic score and both the EDI and daily consumption of fruit and vegetables.

**Table 4:** Top quartile of the Elderly Dietary Index (EDI) and daily fruit and vegetable intake according to adult socio-economic (SE) measures (Odds ratios and 95 % confidence intervals)

	EDI quartiles*	Daily fruit and vegetable intake†
			Unadjusted	Adjusted‡			Unadjusted	Adjusted‡
	*n*	% Q4	OR	95 % CI	*P*	OR	95 % CI	*P*	*n*	%	OR	95 % CI	*P*	OR	95 % CI	*P*
Adult social class																
Non-manual	1875	30·8	1			1			1916	22·1	1			1		
Manual	1936	18·4	0·51	0·44, 0·59	< 0·001	0·66	0·55, 0·79	< 0·001	2036	13·7	0·56	0·47, 0·66	< 0·001	0·65	0·54, 0·79	< 0·001
Education (age at leaving full-time education)																
>14 years	2280	28·8	1			1			2336	20·0	1			1		
≤ 14 years	1209	18·4	0·56	0·47, 0·66	< 0·001	0·61	0·49, 0·76	< 0·001	1264	15·3	0·72	0·60, 0·87	0·001	0·76	0·60, 0·96	0·02
Car ownership																
Yes	3271	26·6	1			1			3368	18·8	1			1		
No	591	13·7	0·44	0·34, 0·56	< 0·001	0·64	0·48, 0·85	0·002	635	13·2	0·66	0·51, 0·84	0·001	0·80	0·60, 1·07	0·13
House ownership																
Yes	3360	26·7	1			1			3464	18·9	1			1		
No	457	11·4	0·35	0·26, 0·48	< 0·001	0·53	0·37, 0·75	< 0·001	494	12·2	0·60	0·45, 0·79	< 0·001	0·77	0·55, 1·08	0·14
Pension																
State+private	2971	27·7	1			1			3053	19·7	1			1		
State only	612	13·1	0·39	0·31, 0·50	< 0·001	0·53	0·40, 0·71	< 0·001	656	10·8	0·50	0·38, 0·64	< 0·001	0·66	0·49, 0·88	0·005
Central heating																
Yes	3517	25·6	1			1			3632	18·5	1			1		
No	253	14·6	0·50	0·35, 0·71	< 0·001	0·67	0·46, 0·99	0·04	266	13·5	0·69	0·48, 0·99	0·05	0·85	0·57, 1·25	0·40
Adverse SE score§																
0	1123	35·4	1			1			1137	24·4	1			1		
1	834	25·9	0·64	0·52, 0·78	< 0·001	0·74	0·59, 0·92	< 0·001	857	18·2	0·69	0·55, 0·86	< 0·001	0·74	0·58, 0·94	< 0·001
2	593	19·7	0·45	0·35, 0·57		0·56	0·42, 0·74		616	14·9	0·55	0·42, 0·71		0·65	0·48, 0·88	
≥ 3	547	12·8	0·27	0·20, 0·35		0·43	0·31, 0·59		580	11·2	0·39	0·29, 0·52		0·50	0·35, 0·71	

* Data for the EDI available for 3924 participants. OR for quartile 4 *v.* quartiles 1–3.

† Data for fruit and vegetable intake available for 4067 participants.

‡ Adjusted for age, energy intake, smoking status, physical activity, alcohol intake, BMI and childhood social class.

§ Score includes manual social class, education ≤ 14 years, no car, not a house owner, state pension only and no central heating.

Examining the combined effects of occupational social class in early and later life showed that diet quality was best in men with both childhood and adult non-manual social class and the poorest in those with both childhood and adult manual social class (Table 5). Exposure to manual social class, whether in childhood or adulthood, was also associated with a poorer diet quality. A test for interaction between childhood and adult social class showed evidence that the effect of childhood social class (manual/non-manual) differed between those of adult manual and non-manual groups (*P*= 0·02 for EDI quartiles). However, no interaction was observed for daily fruit and vegetable intake (*P*= 0·44). There was also a significant inverse trend between the combined adverse childhood and adulthood socio-economic score with both the EDI and daily consumption of fruit and vegetables. Additional sensitivity analyses, examining fruit and vegetable intake separately, showed significant inverse trends with the combined adverse childhood and adulthood socio-economic score, but this association was stronger for vegetables than for fruit consumption.

**Table 5:** Top quartile of the Elderly Dietary Index (EDI) and daily fruit and vegetable intake according to combined childhood and adult socio-economic (SE) measures (Odds ratios and 95 % confidence intervals)

	EDI quartiles*	Daily fruit and vegetable intake†
			Unadjusted	Adjusted‡			Unadjusted	Adjusted‡
	*n*	% Q4	OR	95 % CI	*P*	OR	95 % CI	*P*	*n*	%	OR	95 % CI	*P*	OR	95 % CI	*P*
Childhood/adult social class					< 0·001			< 0·001					< 0·001			< 0·001
Non-manual/non-manual	764	34·2	1			1			775	23·7	1			1		
Manual/non-manual	978	28·7	0·78	0·63, 0·95		0·84	0·68, 1·05		1002	20·8	0·84	0·67, 1·05		0·84	0·67, 1·06	
Non-manual/manual	271	28·0	0·75	0·55, 1·02		0·92	0·66, 1·27		279	17·9	0·70	0·50, 0·99		0·74	0·51, 1·07	
Manual/manual	1454	17·1	0·40	0·33, 0·49		0·49	0·39, 0·61		1529	13·1	0·48	0·39, 0·60		0·53	0·42, 0·67	
Combined adverse childhood and adult SE score§					< 0·001			< 0·001					< 0·001			< 0·001
0–2	905	34·9	1			1			916	23·1	1			1		
3–4	906	26·9	0·69	0·56, 0·84		0·74	0·60, 0·91		927	19·9	0·82	0·66, 1·03		0·81	0·64, 1·03	
5	428	21·5	0·51	0·39, 0·67		0·58	0·44, 0·77		446	15·5	0·61	0·45, 0·82		0·64	0·47, 0·88	
≥ 6	629	15·1	0·33	0·26, 0·43		0·44	0·33, 0·59		661	12	0·45	0·34, 0·60		0·51	0·37, 0·69	

* Data for the EDI available for 3924 participants. OR for quartile 4 *v.* quartiles 1–3.

† Data for fruit and vegetable intake available for 4067 participants.

‡ Adjusted for age, energy intake, smoking status, physical activity, alcohol intake and BMI.

§ Score includes childhood SE measures (childhood manual social class, no bathroom, no hot water supply and no family car ownership) and adult SE measures (manual social class, education ≤ 14 years, no car, not a house owner, state pension only and no central heating).

Table 6 presents the OR of being in the top quartile of the EDI and of consuming fruit and vegetables daily according to the measures of social relationships. Compared with married men, men who were widowed or divorced/separated were significantly less likely to eat fruit and vegetables daily. Men living alone were significantly less likely to be in the highest EDI quartile (OR 0·71, 95 % CI 0·53, 0·95) and to eat fruit and vegetables daily (OR 0·61, 95 % CI 0·44, 0·85) compared with those living with others. However, social contact with children, siblings, friends or neighbours showed no associations with the EDI or daily fruit and vegetable consumption.

**Table 6:** Top quartile of the Elderly Dietary Index (EDI) and daily fruit and vegetable intake according to social relationships (Odds ratios and 95 % confidence intervals)

		EDI quartiles*	Daily fruit and vegetable intake†
		Unadjusted	Adjusted‡			Unadjusted	Adjusted‡
*n*	% Q4	OR	95 % CI	*P*	OR	95 % CI	*P*	*n*	%	OR	95 % CI	*P*	OR	95 % CI	*P*
Marital status																
Married	3224	26·3	1			1			3332	19·2	1			1		
Single	131	16·0	0·53	0·33, 0·86	0·01	0·69	0·42, 1·16	0·16	142	13·4	0·65	0·40, 1·07	0·09	0·66	0·38, 1·15	0·14
Widowed	280	16·8	0·57	0·41, 0·78	0·001	0·70	0·49, 1·00	0·05	292	9·6	0·45	0·30, 0·67	< 0·001	0·53	0·35, 0·80	0·003
Divorced/separated	157	16·6	0·56	0·36, 0·85	0·007	0·65	0·41, 1·04	0·07	166	10·2	0·48	0·29, 0·80	0·01	0·42	0·23, 0·76	0·004
Living alone																
No	3386	25·9	1			1			3497	18·8	1			1		
Yes	423	17·0	0·59	0·45, 0·76	< 0·001	0·71	0·53, 0·95	0·02	450	12·0	0·59	0·44, 0·79	< 0·001	0·61	0·44, 0·85	0·003
Social contact – children																
Every week	3082	24·5	1			1			3184	17·5	1			1		
Every month	217	30·9	1·37	1·02, 1·85	0·78	1·17	0·84, 1·63	0·54	224	22·3	1·35	0·97, 1·88	0·62	1·19	0·83, 1·70	0·80
Every few months to every year	95	29·5	1·29	0·82, 2·01		1·49	0·91, 2·45		100	13·0	0·70	0·39, 1·27		0·71	0·38, 1·33	
Rarely/never/does not apply	210	22·4	0·89	0·63, 1·24		0·96	0·66, 1·39		215	19·1	1·11	0·78, 1·58		1·10	0·75, 1·62	
Social contact – siblings																
Every week	990	23·5	1			1			1036	17·7	1			1		
Every month	680	24·3	1·04	0·83, 1·31	0·16	0·89	0·70, 1·14	0·31	699	18·3	1·04	0·81, 1·34	0·90	0·98	0·75, 1·27	0·80
Every few months to every year	787	26·4	1·17	0·94, 1·45		1·07	0·85, 1·36		810	17·4	0·98	0·77, 1·25		0·92	0·71, 1·20	
Rarely/never/does not apply	687	25·9	1·14	0·91, 1·42		1·09	0·85, 1·40		709	18·2	1·04	0·81, 1·32		0·99	0·76, 1·30	
Social contact – friends																
Every week	3247	25·0	1			1			3357	17·9	1			1		
Every month	246	28·9	1·22	0·91, 1·62	0·70	1·12	0·82, 1·52	0·34	253	18·2	1·02	0·73, 1·42	0·36	0·97	0·68, 1·38	0·33
Every few months to every year	79	30·4	1·31	0·81, 2·13		1·43	0·85, 2·41		84	19·1	1·08	0·62, 1·87		1·20	0·68, 2·11	
Rarely/never/does not apply	55	18·2	0·67	0·33, 1·33		0·92	0·43, 1·96		60	23·3	1·39	0·76, 2·55		1·46	0·73, 2·92	
Social contact – neighbours																
Every week	3306	24·9	1			1			3425	18·1	1			1		
Every month	211	25·1	1·01	0·73, 1·40	0·58	0·87	0·62, 1·23	0·60	217	13·8	0·72	0·49, 1·08	0·70	0·62	0·41, 0·95	0·35
Every few months to every year	57	36·8	1·76	1·02, 3·03		1·76	0·97, 3·20		59	23·7	1·40	0·77, 2·58		1·25	0·64, 2·42	
Rarely/never/does not apply	91	23·1	0·91	0·55, 1·48		1·01	0·59, 1·73		95	16·8	0·91	0·53, 1·58		0·87	0·47, 1·60	

* Data for the EDI available for 3924 participants. OR for quartile 4 *v.* quartiles 1–3.

† Data for fruit and vegetable intake available for 4067 participants.

‡ Adjusted for age, energy intake, smoking status, physical activity, alcohol intake, BMI and adult social class.

## Discussion

The present study examined the associations between a range of childhood and adult socio-economic factors and measures of social relationships with diet quality, assessed by daily fruit and vegetable consumption and the EDI, in older British men aged 60–79 years. The present results show that both childhood and adult socio-economic factors were independently associated with diet quality, with adult factors appearing to be more influential than childhood factors. Diet quality was also influenced by marital status and living arrangements, but showed no association with social contact. The present study adds to the limited literature on the influence of different types of material socio-economic conditions particularly in childhood, and the use of multiple disaggregated socio-economic measures on diet quality in the elderly. The results show that, at least for men, father's social class persists as a strong influence on diet quality in older ages.

We found strong associations between several adult socio-economic measures and diet quality in older men, which were independent of behavioural factors and childhood social class. The magnitude of associations with the EDI was strongest jointly for home ownership and pension (two strong markers of material wealth), followed by education, then car ownership, then social class and then central heating. By contrast, associations with daily fruit and vegetable intake specifically were strongest for social class, then pension and then education. These socio-economic gradients in diet quality are consistent with previous literature showing a healthier diet (characterised by a high intake of fruit, vegetables and other Mediterranean-style food groups) in higher socio-economic groups, measured by occupation, education, income, house ownership or car access in both middle-aged^(^ ^11^ ^,^ ^12^ ^,^ ^14^ ^,^ ^15^ ^,^ ^42^ ^)^ and older adult populations^(^ ^43^ ^–^ ^47^ ^)^. The EDI score specifically previously showed that those in the highest EDI tertile had better financial status and a higher educational level^(^ ^26^ ^)^. The present study extends previous findings by including socio-economic measures particularly relevant to older adults (pension status and central heating) and uses a combination of different adverse adult socio-economic factors, which showed a strong inverse trend with both the EDI score and daily fruit and vegetable intake.

We also found that childhood occupational social class was strongly associated with diet quality, with men of manual father's social class less likely to be in the top EDI quartile and to consume fruit and vegetables daily, independent of behavioural factors and adult social class. With regard to childhood household amenities, there was a borderline significant association between family car ownership and the EDI, which may be due to car ownership being a strong marker of SEP or material wealth, particularly for the generation of this cohort. However, hot water supply and the presence of a bathroom in the house were not associated with diet quality. These findings are consistent with previous studies showing that father's social class influences dietary intake in middle-aged populations^(^ ^16^ ^,^ ^17^ ^,^ ^48^ ^)^. However, one previous study in early old age (61–80 years) showed that childhood social circumstances (social class and per capita household food expenditure) were not strongly related to adult diet quality, as measured by the Healthy Diet Score^(^ ^45^ ^)^. To our knowledge, our findings are the first to confirm that the influences of childhood social class on diet quality in middle-aged populations persist in older ages.

In addition, combining childhood and adult socio-economic factors showed that there were cumulative effects of adverse childhood and adult socio-economic factors on diet quality in older age. This supports previous research in a British adult population suggesting that although adult dietary patterns are determined by childhood influences, diet can be modified as a result of social transition in adulthood^(^ ^17^ ^)^. Previous studies have found that adult socio-economic measures are more influential than childhood socio-economic measures (based on father's occupation or mother's education) on adult diet quality^(^ ^16^ ^,^ ^45^ ^,^ ^49^ ^)^. The present study supports this notion, but in an older adult population, as the magnitude of the effect on diet quality observed for adult occupational social class was greater than that for childhood occupational social class.

Examining the combined effects of occupational social class in early and later life showed that diet quality was best in men with both childhood and adult non-manual social class and the poorest in those with both childhood and adult manual social class (Table 5). Exposure to manual social class, whether in childhood or adulthood, was also associated with a poorer diet quality.

The present results showed that better diet quality in older men is associated with not living alone and being married. This is consistent with previous studies showing that these measures of social relationships are important determinants of diet in the elderly^(^ ^19^ ^,^ ^20^ ^,^ ^46^ ^,^ ^50^ ^,^ ^51^ ^)^, and research has suggested that barriers to healthy eating in older men living alone include poor cooking skills and low motivation to change eating habits^(^ ^52^ ^)^. The literature has also shown that diet quality in older adults is affected by frequency of social contact^(^ ^19^ ^,^ ^20^ ^)^. However, we found no such association in the present study. This may be because most men in this cohort were not socially isolated; the majority of participants had contact with their children, siblings, friends or neighbours at least once per week. More refined categories of social contact may have been needed to identify associations with diet quality.

The major strength of the present study is that it is a large population-based cohort, assessing a range of socio-economic measures, and the analysis has included adjustment for several potentially important confounding factors. However, misclassification of childhood socio-economic status is possible with participants, from lower SEP in particular, overestimating the social class of their father^(^ ^53^ ^)^. This recall bias could have resulted in a weakened association between childhood socio-economic status and diet quality. Dietary intakes were assessed using an eighty-six-item FFQ that has been validated previously against weighed food intakes in the British population^(^ ^30^ ^,^ ^31^ ^)^. The FFQ method is more prone to measurement error than other measures such as weighted food records or 24 h dietary recalls. The collection of dietary data may also have been subject to social desirability bias, and it is possible that low socio-economic groups could have been more affected by this, leading to an underestimation of associations. In elderly populations, in particular, non-response to questions could have increased the chance of under-reporting^(^ ^54^ ^,^ ^55^ ^)^; however, this misclassification is likely to have been non-differential and hence may have biased the results towards the null. Observed associations between socio-economic indicators and diet quality were generally stronger based on the EDI score compared with daily fruit and vegetable intake. This may indicate that a high EDI score is a better marker of an overall healthy diet than using the simpler measure of daily fruit and vegetable consumption in this older population. We examined older men, of predominantly white European ethnic origin. The results are, therefore, limited to this population and should not be applied to women, due to sex differences in dietary intake^(^ ^56^ ^,^ ^57^ ^)^. Further research is needed to replicate findings in other populations. Some residual confounding is possible due to the self-reported nature of variables such as smoking status, alcohol intake and physical activity. Lastly, it is possible that diet quality could also be influenced by additional confounders such as health status, dentition and whether men lived in rural or urban environments. However, adjustment for poor self-reported health made very minor differences to the results. It is possible that both residual and unmeasured confounding may have underestimated or exaggerated the measures of the association observed.

Diet quality in older men is independently influenced by socio-economic factors both in childhood and adulthood, with adult SEP being more influential than early-life SEP in determining dietary patterns. In addition, diet quality is influenced by marital status and adult living arrangements. Public health interventions aimed at improving diet quality of older people need to consider both early- and later-life social circumstances.

## Supplementary material

For supplementary material accompanying this paper visit http://dx.doi.org/10.1017/S0007114515000604. click here to view supplementary material
