# Supplementary material for: Diet quality in older age: the influence of childhood and adult socio-economic circumstances
Source: Br J Nutr. 2015 Apr 1;113(9):1441–52. doi: 10.1017/S0007114515000604 (PMC4462157; doi:10.1017/S0007114515000604)
Supplement: Supplementary file 1 [file S0007114515000604sup001.pdf]

Supplementary Table 1. Elderly Dietary Index scoring criteria

Elderly Dietary Index Scoring					
**Component**	**Score = 1**	**Score = 2**	**Score = 3**	**Score = 4**	**Number of line items from the Food Frequency Questionnaire used to compute each component**
Meat	≥3 days/week	Never/rarely	<1 day/week	1-2 days/week	9
Fish/Seafood	Never/rarely	<1 day/week	≥3 days/week	1-2 days/week	3
Legumes	Never/rarely	<1 day/week	≥3 days/week	1-2 days/week	1
Fruit	<1 day/week	1-2 days/week	3-6 days/week	Daily	2
Vegetables	<1 day/week	1-2 days/week	3-6 days/week	Daily	2
Cereals	<1 day/week	1-2 days/week	3-6 days/week	Daily	8
Bread	None	White	White and whole grain	Whole grain	3
Olive oil[a]	Never/Rarely	Tertile 1 of intake	Tertile 2 of intake	Tertile 3 of intake	1
Dairy	Full-fat milk and full-fat cheese	Semi-skimmed milk and full-fat cheese / full-fat milk and low-fat cheese	Skimmed milk and full-fat cheese	Skimmed/Semi-skimmed milk and low-fat cheese	3

[a]The frequency of olive oil consumption was not available so the scoring of this component was modified from the original score used (1 = <1 day/week; 2 = 1-2 days/week; 3 = 3-6 days/week; 4 = daily) to the quantity of weekly consumption (never/rarely consumed and tertiles of weekly consumption).
